# The Shear Mechanisms of Natural Fractures during the Hydraulic Stimulation of Shale Gas Reservoirs

**DOI:** 10.3390/ma9090713

**Published:** 2016-08-23

**Authors:** Zhaobin Zhang, Xiao Li

**Affiliations:** Key Laboratory of Shale Gas and Geoengineering, Institute of Geology and Geophysics, Chinese Academy of Sciences, Beijing 100029, China; zhangzhaobin@mail.iggcas.ac.cn

**Keywords:** hydraulic stimulation, shale gas, complex fracture network, shear fracture, fluid rock coupling system, boundary element method

## Abstract

The shearing of natural fractures is important in the permeability enhancement of shale gas reservoirs during hydraulic fracturing treatment. In this work, the shearing mechanisms of natural fractures are analyzed using a newly proposed numerical model based on the displacement discontinuities method. The fluid-rock coupling system of the model is carefully designed to calculate the shearing of fractures. Both a single fracture and a complex fracture network are used to investigate the shear mechanisms. The investigation based on a single fracture shows that the non-ignorable shearing length of a natural fracture could be formed before the natural fracture is filled by pressurized fluid. Therefore, for the hydraulic fracturing treatment of the naturally fractured shale gas reservoirs, the shear strength of shale is generally more important than the tensile strength. The fluid-rock coupling propagation processes of a complex fracture network are simulated under different crustal stress conditions and the results agree well with those of the single fracture. The propagation processes of complex fracture network show that a smaller crustal stress difference is unfavorable to the shearing of natural fractures, but is favorable to the formation of complex fracture network.

## 1. Introduction

Shale gas production relies heavily on hydraulic fracturing stimulation due to the low permeability of shale gas reservoirs. During the fracturing treatment of shale gas reservoirs, several mechanisms can lead to permeability enhancement [1], including the propagation of hydraulic fractures (HFs), the opening of natural fractures (NFs) and the shear stimulation. Compared with fracture opening, fracture shearing is more important because it is irreversible, i.e., the shear fracture will not close due to the roughness of fracture surfaces after fluid pressure dissipates in the fracture system. In fact, the microseismic events during fracturing treatment are generally only associated with shear fractures [2,3]. Moreover, both experiments and modelling have demonstrated that the slip on pre-existing natural fractures is important to the effectiveness of the hydraulic fracturing in shale gas reservoirs [4].

The shearing of fractures during the fracturing treatment of shale gas reservoirs has been investigated by many researchers. Zhang et al. [5] derived the governing equations and the boundary conditions for equilibrium shear fractures. WillisRichards et al. [6] presented a model in which the amount of shear displacement depends on the fracture shear stiffness and on the amount of “excess” shear stress. Barton [7] showed the non-linear shear mechanisms, including roughness-dependent dilation and permeability enhancement. Chipperfield et al. [8] presented a shear dilation diagnostics tool for evaluating tight gas stimulation treatments. Nagel and Sanchez-Nagel [9] evaluated the shear failure along fracture surfaces as a function of fracture-induced stress and stress shadowing. Zangeneh et al. [10] investigated the relationship between hydraulic fracturing and the triggering of fracture slip. Nagel et al. [11] and Riahi and Damjanac [1] showed that lower injection rate and lower fluid viscosity favor the creation of shear fracture. McClure and Horne [12,13] showed that shear stimulation is the only possible mechanism when bottom hole fluid pressure is slightly less than the minimum principal stress. Despite the variety of works on shear fracturing, the investigation of shearing fracture treatment is far from perfect. There is little work on the shear mechanisms of natural fractures and how these mechanisms relate to the propagation of complex fracture network during the fracturing treatment of shale gas reservoirs.

Numerical modelling is an important tool for the investigation of hydraulic fracturing process. In early stages, the fracturing processes are always simulated by models such as the Perkins-Kern model [14], the Perkins-Kern-Nordgren model [15], the Khristianovich-Geertsma-Deklerk model [16], pseudo-3D models, and planar-3D models [17]. However, the very idealized fracture configuration used in these traditional models is not appropriate for shale gas reservoirs. One of the most important reasons is the well developed natural fracture network in shale gas reservoirs. Complex fracture networks are often formed due to the complex interactions between hydraulic and natural fractures.

The simulation of complex fracture networks is very challenging. The propagation of fracture networks depends on many factors, such as natural fractures, rock properties, in-situ stress, fluid properties, injection rate, etc. To meet the demands of complex fracture network simulation, many classic methods have been developed to simulate the fluid-rock coupling process of hydraulic fracturing—for example, the finite element method [18], extended finite element method [19], discrete element method [20] and mesh-less method [21,22,23,24,25,26]. These models are used to calculate the stress field by considering the opening and sliding of fractures surfaces. The precision and the efficiency of the stress field solving are crucially important to the modelling of the hydraulic fracturing process. However, the physical phenomena is not only complex but also ill conditioned [20]. The simulation domain is often hundreds of meters, whereas the typical fracture aperture is a small fraction of a millimeter. A deformation that is considered to be small “noise” in the solid solver may induce dramatic oscillation of fluid pressure in the flow solver [20].

The displacement discontinuity method (DDM) [27] is another novel method for modelling the fluid-rock coupling system of hydraulic process. First, fracture displacements can be calculated with higher precision because the analytical solution is directly used to calculate the induced stress. Therefore, the fluid-rock coupling processes can be simulated in a very stable way. Second, the grid number is much less than in other methods because the rock matrix is not discretized. Therefore, the fracturing process can be simulated with very high computational efficiency. The propagation of complex fracture networks with hundreds of fractures can be easily simulated by DDM based models [13,28,29,30,31,32,33]. Moreover, based on the traditional DDM, Verde and Ghassemi [34,35] proposed a fast multipole DDM by considering the difference when calculating the induced stress of far field and near field elements. Using this technique, a fracture system with up to 100,000 boundary elements can be simulated on current mainstream computers. In summary, the propagation of fracture networks during fracturing treatment can be well simulated based on DDM.

In this paper, the shear mechanisms of natural fractures due to fluid injection is investigated by a newly developed model based on DDM. The fluid rock coupling process of the model is carefully designed to solve the slipping of fractures. Both the single fracture and the complex fracture network configurations are used to investigate the shearing of natural fractures. The numerical results demonstrated that the slipping of a NF could occur before the NF is affected by fluid and the slipping of NFs is important to the fracturing treatment of shale gas reservoirs.

## 2. Numerical Method

A DDM based model is used to simulate the hydraulic fracturing process. The details of the model have been introduced in our previously published papers [28,29,36]. The following assumptions are used in the model: The domain of rock matrix is infinite and the rock matrix is homogeneous, isotropous and linear elastic [27]. The rock matrix is impermeable. The fluid injected is Newtonian, single phase and laminar [13,37,38].

Given the normal and shear displacement discontinuities (DDs) of each fracture element, the induced stresses by the opening and sliding of the fracture system with *N* elements can be calculated by [38]

(1) $$\begin{array}{l} \sigma_{n}\left(x\right)=\sum_{r=1}^{N}\int_{0}^{l_{r}}\left[G_{11}\left(x,s\right)w\left(s\right)+G_{12}\left(x,s\right)\nu \left(s\right)\right]K\left(x,s\right)ds\\ \tau_{s}\left(x\right)=\sum_{r=1}^{N}\int_{0}^{l_{r}}\left[G_{21}\left(x,s\right)w\left(s\right)+G_{22}\left(x,s\right)\nu \left(s\right)\right]K\left(x,s\right)ds \end{array}$$

where ***x*** = (*x*, *y*) is the coordinate; *w* is the normal DD; *v* is the shear DD; *l_r_* is the length of fracture; *r*, *G_ij_* are the hyper singular Green’s functions, which are proportional to the plane strain Young’s modulus [38]; *σ_n_* is the normal stress and *τ_s_* is the shear stress, obeying Coulomb’s frictional law characterized by the coefficient of friction *λ*, which limits the shear stress by

(2) $$\left|\tau_{s}\right|\leq \lambda \sigma_{n}$$

which can act in parts of fractures that are in contact, but vanishes along the separated parts. Along the opened fracture portions, we have

(3) $$\sigma_{n}=p_{f}$$

*K* is the three dimensional correction coefficient proposed by Olson [39].

The calculation of the shear DD of each fracture element is important and is one of the most difficult processes during the fluid-rock coupling iteration of the model. Most of the difficulties come from the non-linearity of the problem caused by the friction between fracture surfaces. With the increasing of fluid pressure, both the normal and shear stresses along each fracture element change. The slipping of a fracture occurs once the shear stress along the element exceeds a threshold value, specifically, when Equation (2) is not satisfied. However, there are infinite shear DDs that satisfy Equation (2) so that shear DD cannot be calculated directly. Given the in-situ stress condition along a fracture element, the Equation (2) is satisfied when shear DD is in the range

(4) $$\frac{\lambda \sigma_{n}-\left|\tau_{s}\right|}{G_{22}}\leq v\leq \frac{\lambda \sigma_{n}+\left|\tau_{s}\right|}{G_{22}}$$

**Figure 1:**
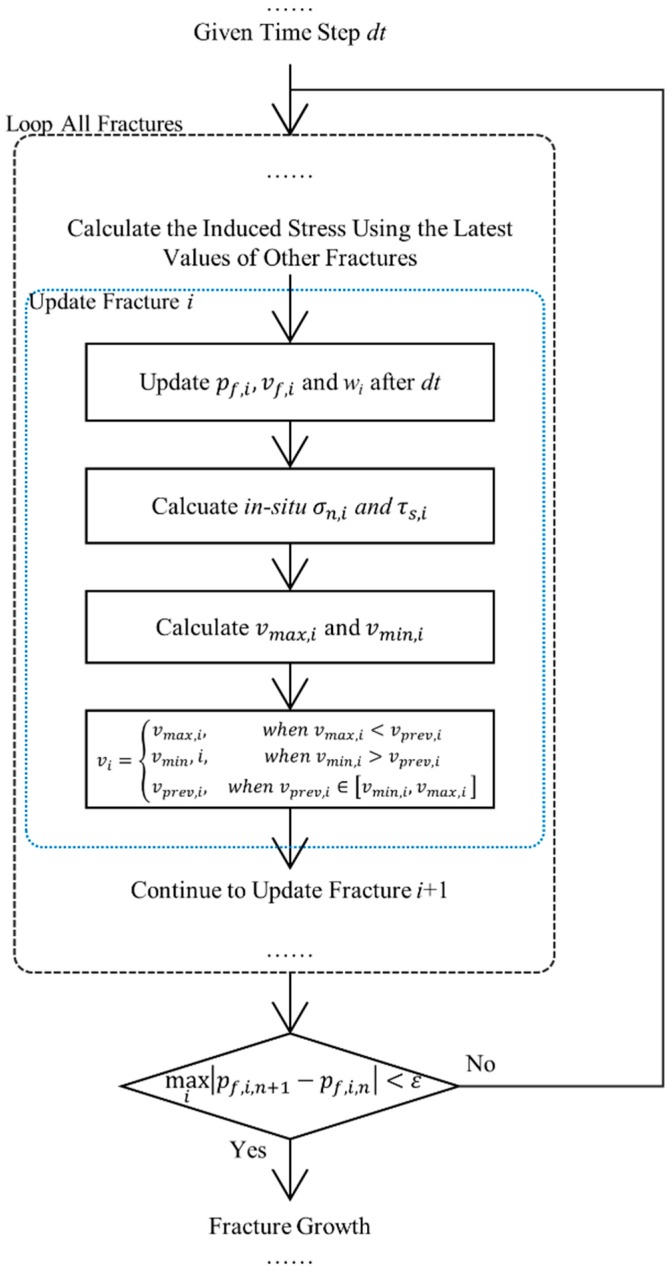
The flowchart for updating the shear displacement discontinuity of each fracture element after the given time step *dt* during the fluid-rock coupling iteration. Here, *p_f_* and *v_f_* refer to fluid pressure and fluid volume, respectively, *v_max_* and *v_min_* are the maximum and the minimum values of shear displacement discontinuities, respectively, and *v_prev_* is the shear displacement discontinuity of the fracture element in the last step.

The value of shear DD in the last step is used to determine the new value. Specifically, we will find the value of shear DD with the minimum difference with the value in the last step within the range defined by Equation (4), i.e., the slipping of fracture surfaces will stop immediately after the Equation (4) is satisfied. During the fluid-rock coupling iteration of the model, the shear DD of each fracture element is recorded so that the fractures may slip within the minimum distance to get the stable states. The flowchart for update the shear DD after the given time step *dt* during the fluid-rock coupling iteration is illustrated in Figure 1.

The fracture growth is based on the maximum hoop stress criterion, with the maximum mixed-mode intensity factor reaching a critical value

(5) $$\frac{1}{2}\cos\frac{\theta_{0}}{2}\left[K_{I}\left(1+\cos\theta_{0}\right)-3K_{II}\sin\theta_{0}\right]=K_{IC}$$

where *K_I_* and *K_II_* are stress intensity factors; *K_IC_* is tensile mode fracture toughness; and θ is the fracture propagation direction relative to the current fracture orientation and satisfies

(6) $$K_{I}\sin\theta +K_{II}\left(3\cos\theta -1\right)=0$$

**Figure 2:**
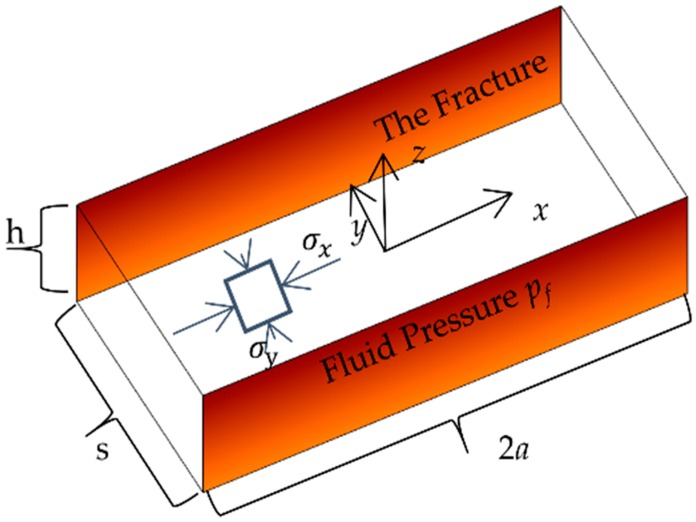
The validation problem of comparing this work to Kresse, Weng, Gu and Wu [30] and FLAC3D: two parallel straight fractures.

**Figure 3:**
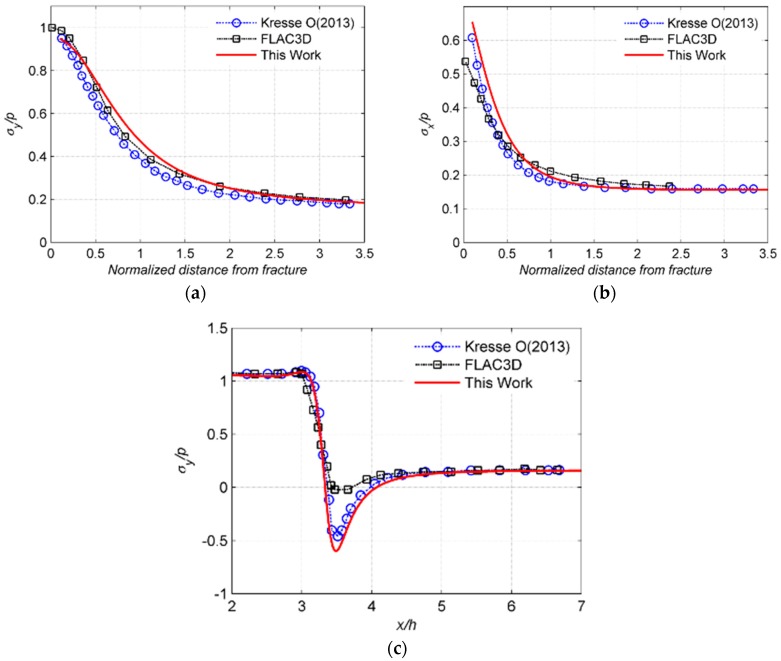
Comparison of the model with FLAC3D and the numerical model of Kresse, Weng, Gu and Wu [30]. (**a**) $\sigma_{y}$ along the *y*-axis; (**b**) $\sigma_{x}$ along the *y*-axis; (**c**) $\sigma_{y}$ along the *x*-axis.

The stress interactions between fractures are important to the propagation of the fracture network as the in-situ stress could be altered by the opening or sliding of fractures. In this work, the fracture interaction between two parallel fractures is validated against FLAC3D and the numerical results of Kresse, Weng, Gu and Wu [30]. The numerical setting is shown in Figure 2. Two parallel straight fractures with finite lengths and heights are simulated. The fracture in FLAC3D is simulated as two surfaces at the same location but with unattached grid points by Kresse, Weng, Gu and Wu [30]. Constant internal fluid pressure is applied to the two fractures. The two fractures have the same length and height with the ratio of height/half-length = 0.3. The two fractures are also closed spaced with *s*/*h* = 0.5, where *s* is fracture spacing and *h* is fracture height. The stresses along *x*-axis (*y* = 0) and *y*-axis (*x* = 0) are compared. The results are shown in Figure 3. The results of this work closely match the previous works.

## 3. Results and Discussion

### 3.1. The Shearing of Single Fracture

**Figure 4:**
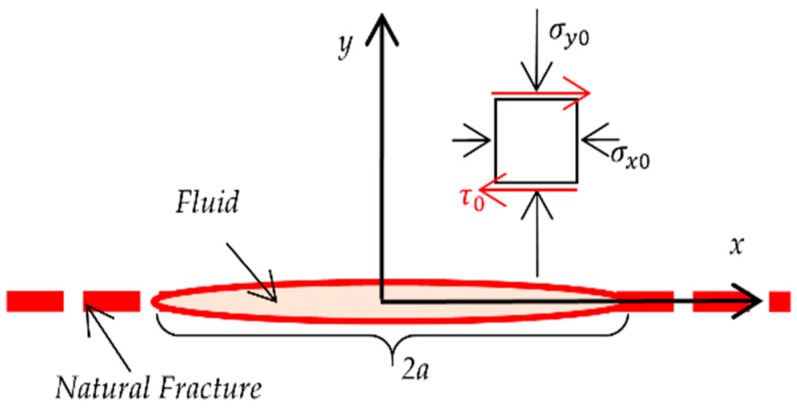
Numerical setting for investigating the shearing of a single natural fracture in an infinite rock matrix. The fracture part between −a≤x≤a is applied with constant internal fluid pressure.

A single fracture in an infinite rock matrix is simulated to show how a hydraulic fracture propagates along natural ones. Considering a single natural fracture along the *x*-axis as shown in Figure 4, part of the fracture has been filled with pressurized fluid. The natural fracture is reopening by the increasing fluid pressure.

**Figure 5:**
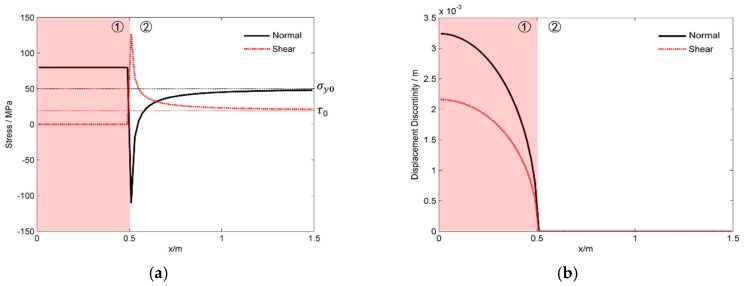
The stress and the displacement discontinuities along the natural fracture when the strength of the natural fracture is assumed to be infinite. (**a**) The normal and shear stresses; (**b**) the normal and shear fracture displacement discontinuities.

Firstly, we exported the fractures states when the strength of the natural fracture is assumed to be infinite, i.e., the DDs equals zero at the fracture parts that are not filled with fluid. The numerical results are shown in Figure 5. The fracture can be clearly divided into two parts. The first part is the fracture section that is filled with fluid. Within the first part, the normal stress is equal to fluid pressure and the shear stress is equal to zero. Both the normal and shear DDs decrease with *x* and equal to zero at the tip of the fluid-filled part (when *x* = 0.5 in Figure 5). The second part is the fracture sections that are neither opened nor slipped, i.e., both the normal and shear DDs equal to zero. The stresses at the points far away from the first part equal to the far field stress. This is caused by the very weak induced stress by the first part. By contrast, the in-situ stress condition is controlled by the DDs of the first part at the points near the first part. As shown in Figure 5a, the stresses near the tip of the fracture shows strong singularity. Both the tensile and shear stresses tend to be infinite near the fracture tip. In fact, the induced stresses of a fracture near the fracture tip are given by the theoretical solutions of elastic mechanics:

(7) $$\sigma_{y}=\frac{K_{I}}{\sqrt{2\pi r}}$$

(8) $$\tau =\frac{K_{II}}{\sqrt{2\pi r}}$$

where $\sigma_{y}$ and τ are normal and shear induced stress respectively; *K_I_* and *K_II_* are the mode I and mode II stress intensity factors respectively; and *r* is the distance to fracture tip.

**Figure 6:**
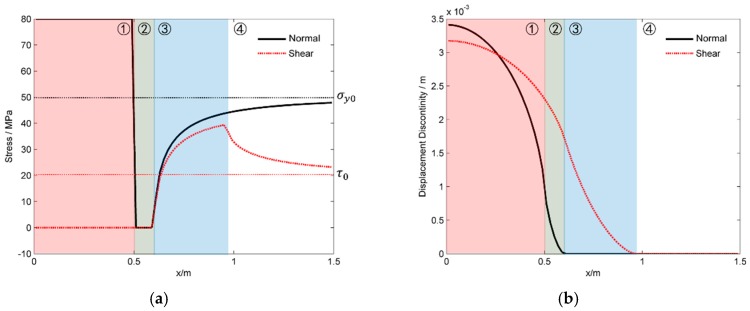
The stress and the displacement discontinuities along the natural fracture when the natural fracture has zero tensile strength. (**a**) The normal and shear stresses; (**b**) the normal and shear fracture displacement discontinuities.

However, neither the tensile stress nor the shear stress could be infinite in the real case. The hydraulic fracture will propagate along the natural one when the in-situ stress exceeds the natural fracture strength. Figure 6 shows the stresses and the DDs along *x*-axis when the natural fracture has zero tensile strength. The parameters of the modelling are listed in Table 1. Different with the case when the strength is assumed to be infinite, the fracture can be divided into four different parts. The first part is the fracture section that is filled by fluid. This part is similar to the results as shown in Figure 5, but both the tensile and shear DDs are not equal to zero at the tip of the fluid-filled part. The main reason relies on the reopening and the slipping of the natural fracture beyond the fluid-filled part. The second part is the natural fracture sections that are reopened but have no fluid filling. The reopening of the second part is caused by the induced tensile stress of the first part, which is clearly shown in Figure 5a. Different from the very high tensile stress in part 2 as shown in Figure 5a, the tensile stress equals to zero in Figure 6a, which is reasonable as the natural fracture part is broken and is no filling with fluid. The third part shown in Figure 6 is the natural fracture sections that are still in contact but have finite slipping distance due to the in-situ shear stress. As the fracture surface is still in contact, the slipping distance increases until the in-situ shear stress could be balanced by the friction stress between the fracture surfaces. The shear stress, which equals the friction stress, increases with *x* because the normal stress increases with *x*. Moreover, the ratio between the shear stress and the normal stress is exactly 0.9, which equals the friction coefficient between the fracture surfaces. The forth part is the section of natural fractures that are neither reopened nor slipped. Both the tensile and shear DDs are equal to zero in this part. Moreover, both the normal and shear stresses tend to be the far-field stress with the increasing of *x* coordinate, as the induced stresses are weaker when the coordinate *x* is bigger.

### 3.2. The Shear Fracture Length

**Figure 7:**
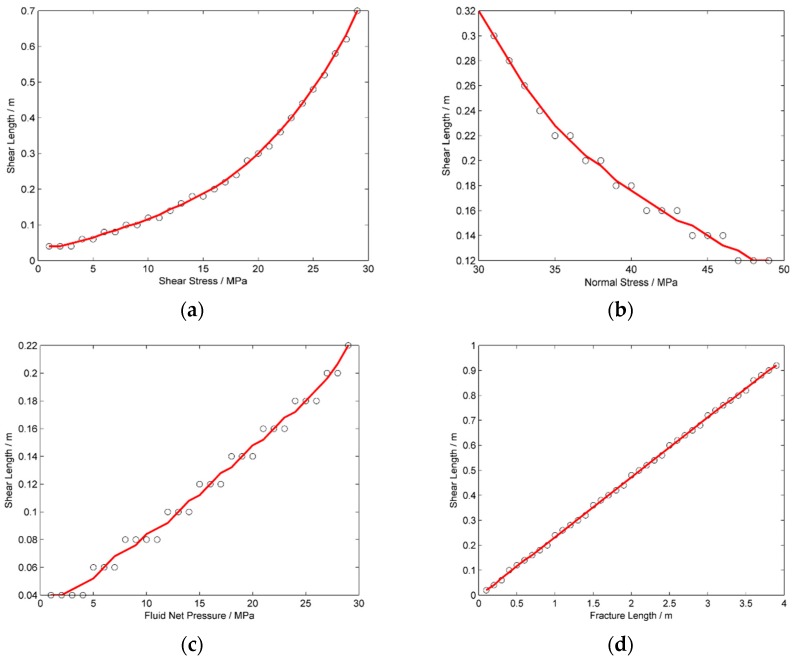
The variations of the length of the shear fracture that is not affected by fluid pressure with (**a**) the shear stress along the natural fracture; (**b**) the normal stress perpendicular with the natural fracture; (**c**) the fluid net pressure and (**d**) the length of the fracture part that is filled by fluid.

From the analysis in Section 3.1, it is clear the surfaces of a natural fracture may slip before the natural fracture is filled by pressurized fluid. The question is, how long is the shear fracture beyond the fluid-filled part? The formation of the shear fracture is meaningful to the permeability enhancement of shale gas reservoir only when the shear fractures have non-ignorable lengths. In this section, we analyzed the variation of shear fracture length, which is defined as the sum length of the second and third parts of the natural fracture as shown in Figure 6, with several environmental parameters. First, the shearing length increases with the shear stress along the natural fracture as shown in Figure 7a. Moreover, the increasing rate of the shearing length increases with the shear stress. This indicates that the slight increasing of shear stress may have great effects on the increasing of shear length when the shear stress is bigger. Second, the shearing length decreases with the normal stress as shown in Figure 7b. This is caused by the fact that the friction stress between the fracture surfaces increases linearly with the contact stress, which is proportional to the normal stress. The slipping distance between the fracture surfaces is shorter when there is greater friction stress that resists the shearing of the fracture surfaces. Thirdly, the shearing length of the natural fracture increase nearly linearly with the fluid net pressure as shown in Figure 7c. This is caused by the fact that the friction stress between the natural fracture surfaces decreases with the increasing of the tensile stress, which increases with the fluid pressure as the induced tensile stress is bigger when the fracture aperture is wider. Finally, the shearing length increases exactly linearly with the length of the fracture parts that are filled by pressurized fluid as shown in Figure 7d. This is caused by the fact that both the tensile stress and the shear stress of a fracture are stronger when the fracture is longer. This result indicates that there is a ratio between the shearing length and the fluid-filled length. This ratio is of great importance to us because it is possible for us to estimate the length of the shear fracture when the length of the fluid-filled region is known.

### 3.3. The Shearing of a Complex Fracture Network

**Figure 8:**
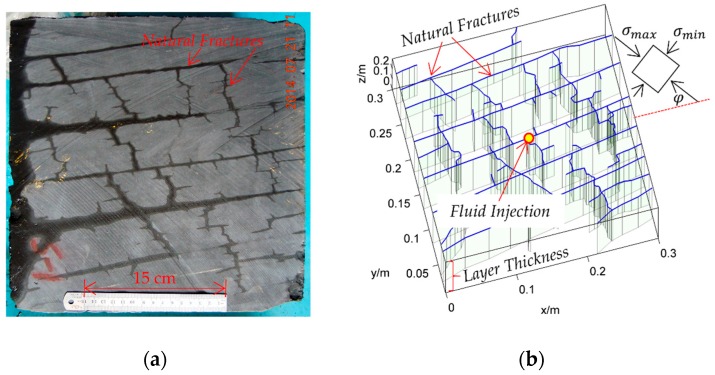
The numerical setting for simulating the propagation of complex fracture network. (**a**) The shale sample from Longmaxi formation of China; (**b**) The natural fracture network reconstructed from the shale sample. The natural fractures are represented by the light faces with blue lines on the top. The fluid is injected from the center of the region.

As the natural fracture network of shale gas reservoirs is always well developed and there are complex interactions between the hydraulic and natural fractures, the real hydraulic fracturing process is much more complex than the idealized fracture configurations analyzed above. Figure 8a shows one of the shale samples from the Longmaxi formation of China. The natural fracture network is well developed. The propagation of a complex fracture network is simulated based on the natural fracture network reconstructed from the shale sample in this section. The reconstructed natural fracture network is shown in Figure 8b, which contains most of the natural fractures in the photo in Figure 8a. The natural fractures can be roughly divided into two sets, the horizontal set and the vertical set. The fractures are longer and the connectivity is better for the horizontal set of fractures. Although most of the main fractures in Figure 8a are reconstructed in Figure 8b, there are many small fractures that are difficult to identify from the photo. In fact, it is impossible to get all the natural fractures. However, the effects of these small fractures cannot be ignored because the strength of a fracture is much lower than the rock matrix. Hydraulic fractures have a greater opportunity to propagate along the natural fractures than through the rock matrix. To simulate the effects of these small fractures, a virtual fracture system is used. A fracture element is treated as virtual when it is not incorporated into the coupled iteration when solving the pressure and displacement discontinuities. For more details on the virtual fracture system, please refer to our previously published papers [28,29,36]. Fluid is injected from the center of the region. The simulation is finished when any fracture propagates outside the current region. The simulation is implemented under two different crustal stress conditions.

**Figure 9:**
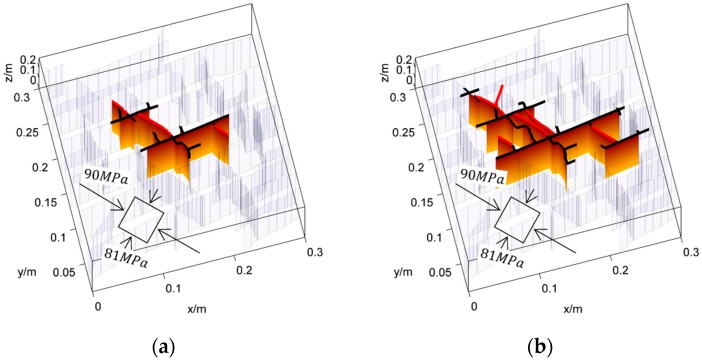

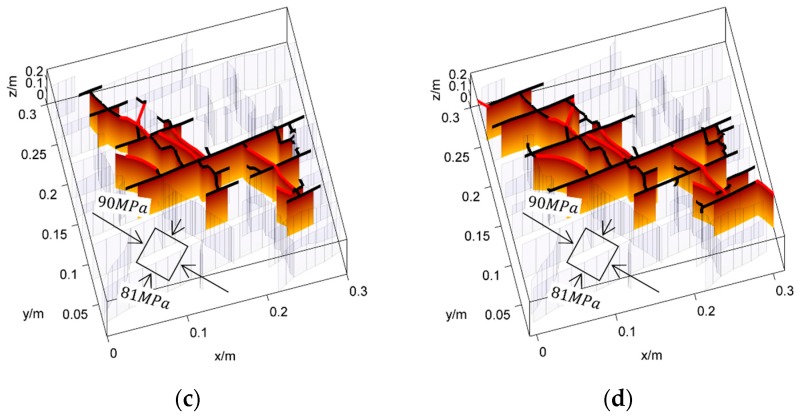
The propagation processes of the hydraulic fractures when the far field stress is $\sigma_{max}=90\;\text{MPa},\;\sigma_{min}=81\;\text{MPa}$. The tensile mode fractures that are affected by fluid pressure are represented by the colored faces. The shearing fractures are marked by the bold black curves at top. The newly formed hydraulic fractures are marked by the bold red curves at top. The unaffected fractures are represented by the light mesh.

**Figure 10:**
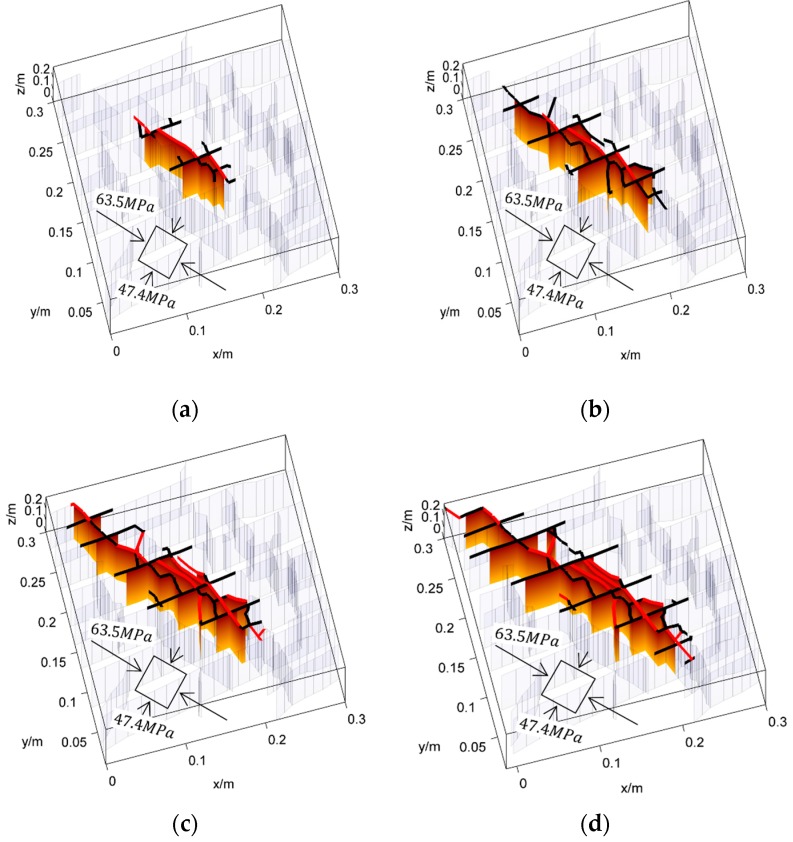
The propagation processes of the hydraulic fractures when the far field stress is $\sigma_{max}=63.5\;\text{MPa},\;\sigma_{min}=47.4\;\text{MPa}$. The tensile mode fractures that are affected by fluid pressure are represented by the colored faces. The newly formed hydraulic fractures are marked by the bold red curves at top. The shearing fractures are marked by the bold black curves at top. The unaffected fractures are represented by the light mesh.

The propagation of a complex fracture network is first simulated under the crustal stress $\sigma_{max}=90\;\text{MPa},\;\sigma_{min}=81\;\text{MPa}$ at a depth of 4200 m in Longmaxi formation of southern China, where $\sigma_{max}$ and $\sigma_{min}$ are the maximum and the minimum principle stress respectively. The more details of the input parameters are listed in Table 2. The fracture network propagation processes are shown in Figure 9. The fracture network propagates mainly along the natural fractures and the maximum principle stress direction. As the crustal stress is as big as 90 MPa and the stress difference is only 9 MPa, the shear fractures are not well developed. The main reason is the very high normal stress perpendicular to the fractures suppressing the slipping of fracture surfaces. However, the shearing of fractures could also be identified beyond the tip of the fracture sections that are filled by fluid. For the sake of comparison, the fracture network propagation processes are also simulated under the crustal stress of $\sigma_{max}=63.5\;\text{MPa},\;\sigma_{min}=47.4\;\text{MPa}$ at a depth of 2380 m. The results are shown in Figure 10. It can be seen that the propagation processes are similar to that shown in Figure 9. The hydraulic fractures propagates mainly along natural fractures and the maximum principle stress direction. However, there are also significant differences. First, as the crustal stress is much lower at 2380 m depth than at 4200 m, the shearing of fractures is less suppressed by the normal stress. Moreover, the stress difference is about 16 MPa at the depth of 2380 m, which is much bigger than that of 4200 m. As a result, the shearing of fractures is much more significant. Comparing the results shown in Figure 10 with that in Figure 9, it is clear that the shearing fracture lengths beyond the fluid-filled parts are much longer than that in 4200 m. The second difference is the shape of the affected region. As the anisotropy of crustal stress is much stronger in 2380 m, the propagation distance along the natural fractures is much shorter than that in 4200 m. Therefore, the affected area is much smaller. Despite the differences between these two cases, we could conclude from the propagation of complex fracture network that the shearing of natural fractures before being affected by fluid is a common occurrence.

From the results of a single fracture and complex fracture network shown in the above sections, it is found that the propagation mechanisms of a natural fracture are much different from those of newly formed fractures. For the newly formed fractures, the shearing is always ignorable. The most important reason is the changing of propagation direction. The propagation direction of hydraulic fractures could change until the shear stress along the fracture equals to zero. The direction change of hydraulic fractures can be seen in the propagation of complex fracture network shown in Figure 9 and Figure 10. However, if natural fractures exists, the direction change is highly suppressed. The hydraulic fractures will propagate along the natural ones in most of the cases. As a result, the shear stress always exists along the natural fractures. The induced shear stress caused by the slipping of fracture surfaces is important in balancing the in-situ shear stress. As a result, the shearing of natural fracture before affected by fluid could happen in most of the cases. From the results of single fracture, it can be seen that the shearing length is not ignorable even when the shearing stress is only 1 MPa. Therefore, for the naturally fractured shale samples, shear strength is much more important than the tensile mode strength.

## 4. Conclusions

The shearing of natural fractures during the hydraulic fracturing treatment of shale gas reservoirs are investigated by a newly proposed model based on the displacement discontinuities method. The analyses are implemented based on both the single natural fracture and the complex fracture network reconstructed from the shale sample of Longmaxi formation of China.

The shearing of a natural fracture exists in most cases and the shearing of a natural fracture before being filled by fluid has a non-ignorable length. Therefore, for the naturally fractured shale samples, shear strength is much more important that the tensile mode strength.

A ratio exists between the lengths of shearing fracture and fracture parts that are filled by pressurized fluid. Therefore, the shearing of natural fractures is more important during the later stages of hydraulic fracturing treatment.

A natural fracture can be divided into four parts: the fluid-filled part, the tensile and shear part, the pure shear part and the contact part.

There is no stress singularity at the tip of the fluid-filled natural fractures due to reopening and the slipping of fractures surfaces along the natural fracture beyond the fluid-filled part. The normal stress near the tip of the fluid-filled part is smaller than the far field normal stress. The shear stress is restricted by the product of normal stress and the friction coefficient between fracture surfaces.

The smaller crustal stress different is unfavorable to the shearing of natural fractures, but is favorable to the formation of complex fracture network as the propagation distance is farther along the minimum principle stress direction when the stress difference is smaller.

## Figures and Tables

**Table 1:** Input parameters for simulating the shearing of a single fracture. Here, *a* refers to the half-length of the fracture section that is filled by pressurized fluid.

Parameter	Value	Parameter	Value
*a*	0.5 m	Layer thickness	Infinite
Fluid Net Pressure	30 MPa	$\sigma_{y0}$	50 MPa
$\tau_{0}$	20 MPa	Young’s Modulus	$1.8\times 10^{10}$ Pa
Poisson’s ratio	0.1	Friction Coefficient	0.9

**Table 2:** Input parameters for simulating the propagation of complex fracture network. Here *K_IC_* refers the mode I stress intensity factor of rock and φ is the angle between the maximum principle stress direction and the negative *x*-axis direction as illustrated in Figure 8b.

Parameter	Value	Parameter	Value
Injection Rate	$1.0\times 10^{-3\;}m^{2}/s$	Layer thickness	0.3 m
Fluid Viscosity	0.1 cP	Friction Coefficient	0.9
Young’s Modulus	$1.8\times 10^{10}\;\text{Pa}$	Poisson’s ratio	0.1
*K_IC_*	1.0 × 10^6^ Pa·m^0.5^	φ	45°
